# Supplementary material for: Distinct Plasma Concentrations of Acyl-CoA-Binding Protein (ACBP) in HIV Progressors and Elite Controllers
Source: Viruses. 2022 Feb 23;14(3):453. doi: 10.3390/v14030453 (PMC8949460; doi:10.3390/v14030453)
Supplement: Supplementary file 1 [file viruses-14-00453-s001.zip › Viruses-ACBP-Supplementary-22Feb2022.pdf]

Table S1. Characteristics of study participants.

		EC	HIV ART+	HIV ART-	Uninfected controls	P value*
**n**		**37**	**55**	**27**	**31**	
Age	Median (min-max)	45.5 (25-72)	54 (26-74)	34 (21-60)	52 (23-75.5)	<0.001
Sex	Female n (%)	9 (24%)	5 (9%)	3 (11%)	8 (26%)	0.09
	Male n (%)	28 (86%)	50 (91%)	24 (89%)	23 (74%)	
HIV viral load (log_{10} copies/mL)	Median (min-max)	<50 (<50-<20)	<50 (<50-<20)	4.7 (2.8-5.9)	NA	NA
Duration of infection (years)	Median (min-max)	6 (0.7-27)	17.2 (2-33)	0.3 (0.1-13)	NA	<0.0001
Duration of treatment (years)	Median (min-max)	NA	13.7 (0.2-25.4)	NA	NA	NA
CD4 T-cell count	Median (min-max)	640 (290-1200)	546 (1.4-1462)	310 (57-910)	827 (281-1173)	<0.0001
CD8 T-cell count	Median (min-max)	649 (211-1460)	719 (2.46-1475)	900 (300-2832)	391 (188-1245)	0.0002
CD4/CD8	Median (min-max)	1.02 (0.34-3.06)	0.7 (0.2-2.1)	0.4 (0.05-1.2)	2.1 (0.38-3.98)	<0.0001

*Anova test.

Table S2. HLA types and plasma ACBP levels of HIV elite controller participants

	HLA-A alleles		HLA-B alleles		HLA-C alleles		Protective HLA	Plasma ACBP (ng/mL)
1	A*03:01	A*11:01	B*15:01	B*35:01	C*03:03	C*12:03	No	71,5
2	A*02:01	A*30:01	B*07:02	B*42:01	C*04:01	C*17:01	No	91,8
3	A*02:05	A*74:02	B*27:03	B*49:01	C*02:02	C*07:01	Yes	80,0
4	A*30:02	A*34:02	B*14:02	B*44:03	C*04:01	C*08:02 OR C*05:01	No	259,2
5	A*34:02	A*66:01	B*53:01	B*58:01	C*04:01	C*07:01	Yes	74,1
6	A*02:02	A*30:01	B*57:03	B*58:02	C*06:02	C*17:01	Yes	58,6
7	A*02:01	A*03:01	B*07:02	B*18:01	C*07:01/ 07:02	C*07:02	No	155,0
8	A*03:01	A*30:02	B*27:03	B*51:01	C*07:01	C*16:01	Yes	142,4
9	A*68:02	A*74:02	B*15:10	B*41:01	C*03:04	C*07:01	No	179,8
10	A*02:01	A*02:01	B*44:02	B*44:02	C*05:01	C*05:01	No	28,7
11	A*02:01	A*32:01	B*40:01	B*40:01	C*03:04	C*03:04	No	119,9
12	A*02:05	A*24:02	B*14:02	B*40:02	C*03:05	C*08:02	No	160,0
13	A*03:01	A*24:02	B*07:02	B*27:01	C*07:02	C*07:02	Yes	70,1
14	A*01:01	A*03:01	B*08:01	B*14:02	C*07:01	C*08:02	No	59,4
15	A*01:01	A*32:01	B*27:03/ 27:05	B*57:02	C*01:02/ 01:03	C*18:01/ 18:02	Yes	109,8
16	A*02:01	A*02:01	B*27:05	B*40:02	C*02:02	C*15:02	Yes	254,7
17	A*03:01	A*24:02	B*14:02	B*27:05	C*02:02	C*08:02	Yes	166,3
18	A*02:01	A*11:01	B*08:01	B*56:01	C*01:02	C*07:01	No	113,1
19	A*03:01	A*68:02	B*53:01	B*53:01	C*02:02	C*04:01	No	169,0

20	A*24:03	A*32:01	B*18:01	B*44:02	C*05:01/ 05:02/ 05:03/ 05:04/ 05:05 OR C*08:02	C*12:03/4	No	203,4
21	A*02:01	A*26:01	B*07:02	B*38:01	C*07:02	C*12:03	No	100,0
22	A*29:02	A*33:03	B*44:03	B*81:01 OR 81:02	C*02:02	C*07:01	No	110,4
23	A*01:01	A*03:01	B*13:02	B*35:03	C*04:01	C*06:02	No	131,9
24	A*02:01	A*02:06	B*27:05	B*44:02	C*02:02	C*02:02	Yes	189,3
25	A*02:01	A*03:01	B*07:02 OR B*48:08	B*48:01 OR B*81:01/ 81:02	C*07:02	C*08:03	No	149,4
26	A*01:01	A*03:01	B*07:02	B*57:01	C*06:02	C*07:02	Yes	109,8
27	A*02:01	A*03:01	B*14:02	B*44:02	C*05:01	C*08:02	No	126,4
28	A*02:02	A*33:03	B*35:01	B*57:01	C*12:03	C*18:01	Yes	72,8
29	A*02:01	A*24:02	B*15:01	B*27:05	C*02:02	C*03:03	Yes	72,1
30	A*02:01	A*25:01 OR A*26:03	B*18:01	B*27:05	C*01:02	C*12:03	Yes	43,8
31	A*02:01	A*68:01	B*15:01	B*27:05	C*02:02	C*03:03	Yes	97,6
32	A*02:01	A*11:01	B*40:06	B*51:01	C*14:02	C*15:02	No	32,9
33	A*02:01	A*30:01	B*13:02	B*57:01	C*06:02	C*06:02	Yes	129,4
34	A*11:01	A*24:02	B*51:01	B*54:01	C*01:02	C*14:02	No	85,5
35	A*01:01	A*24:02	B*57:01	B*57:01	C*06:02	C*06:02	Yes	34,9
36	A*01:01	A*03:01	B*08:01	B*14:02	C*07:01	C*08:02	No	85,5
37	A*02:01	A*68:02	B*51:01	B:57:02	C:14:02	C*18:02	Yes	69,5

Protective HLA: B*27, B*57, B*58

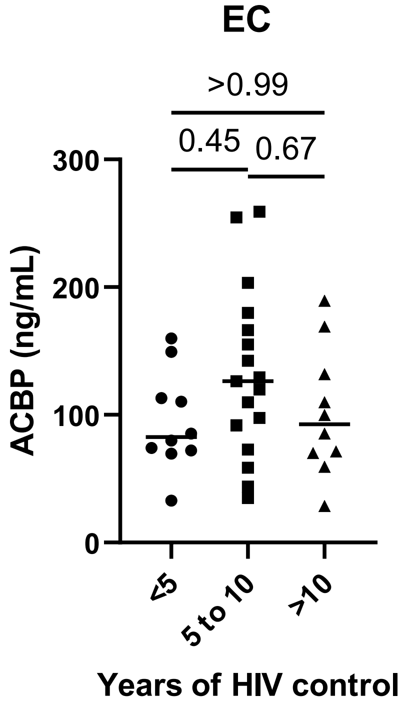

Figure S1: years of HIV control did not influence ACBP levels in HIV elite controllers (EC). Kruskal-wallis's test with Dunn's post-test.

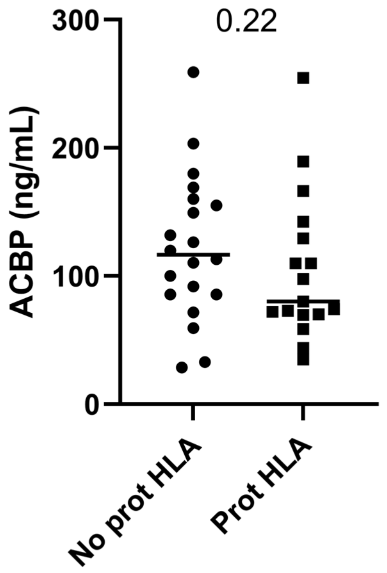

Figure S2. plasma ACBP levels in EC harboring or not protective (prot) HLA -B*27, -B*57 or -B*58. Mann-Whitney's test.

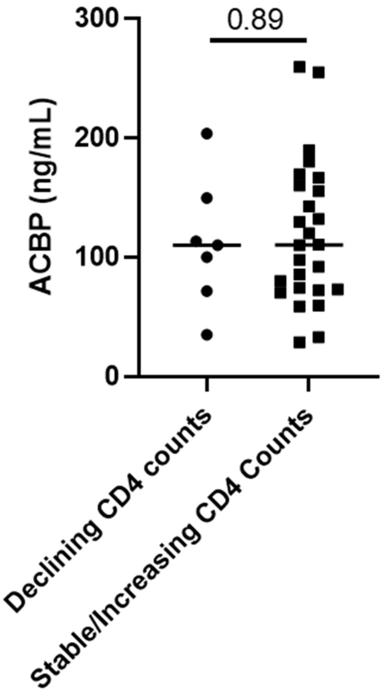

Figure S3. plasma ACBP levels in EC with CD4 decay (Declining CD4 count) or not (Stable/increasing CD4 counts). Mann-Whitney's test.

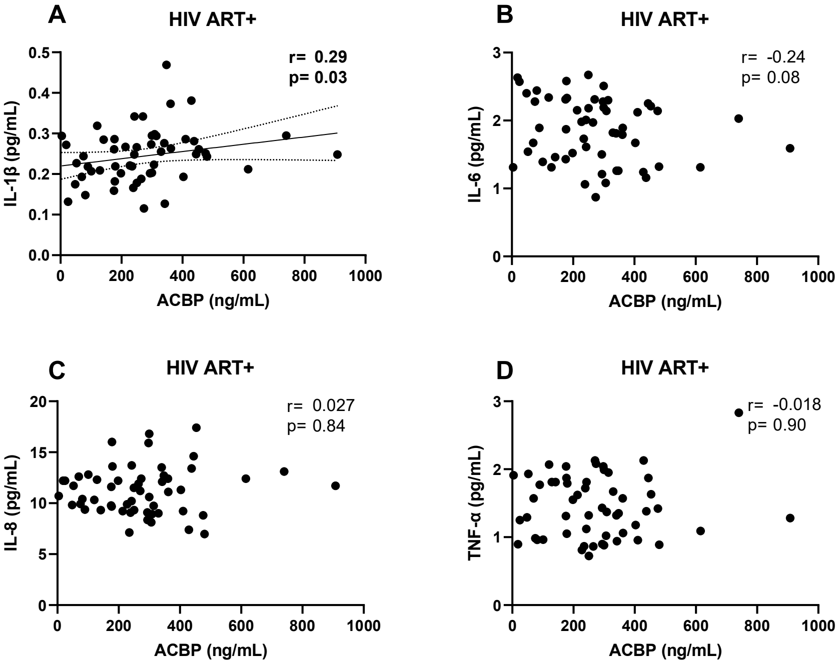

Figure S4. correlations between plasma levels of ACBP and IL-1β, IL-6, IL-8 and TNF-α in ART-treated PLWH. Spearman's test.
