# Supplementary figures and images for: Distinct Plasma Concentrations of Acyl-CoA-Binding Protein (ACBP) in HIV Progressors and Elite Controllers
Source: Viruses. 2022 Feb 23;14(3):453. doi: 10.3390/v14030453 (PMC8949460; doi:10.3390/v14030453)

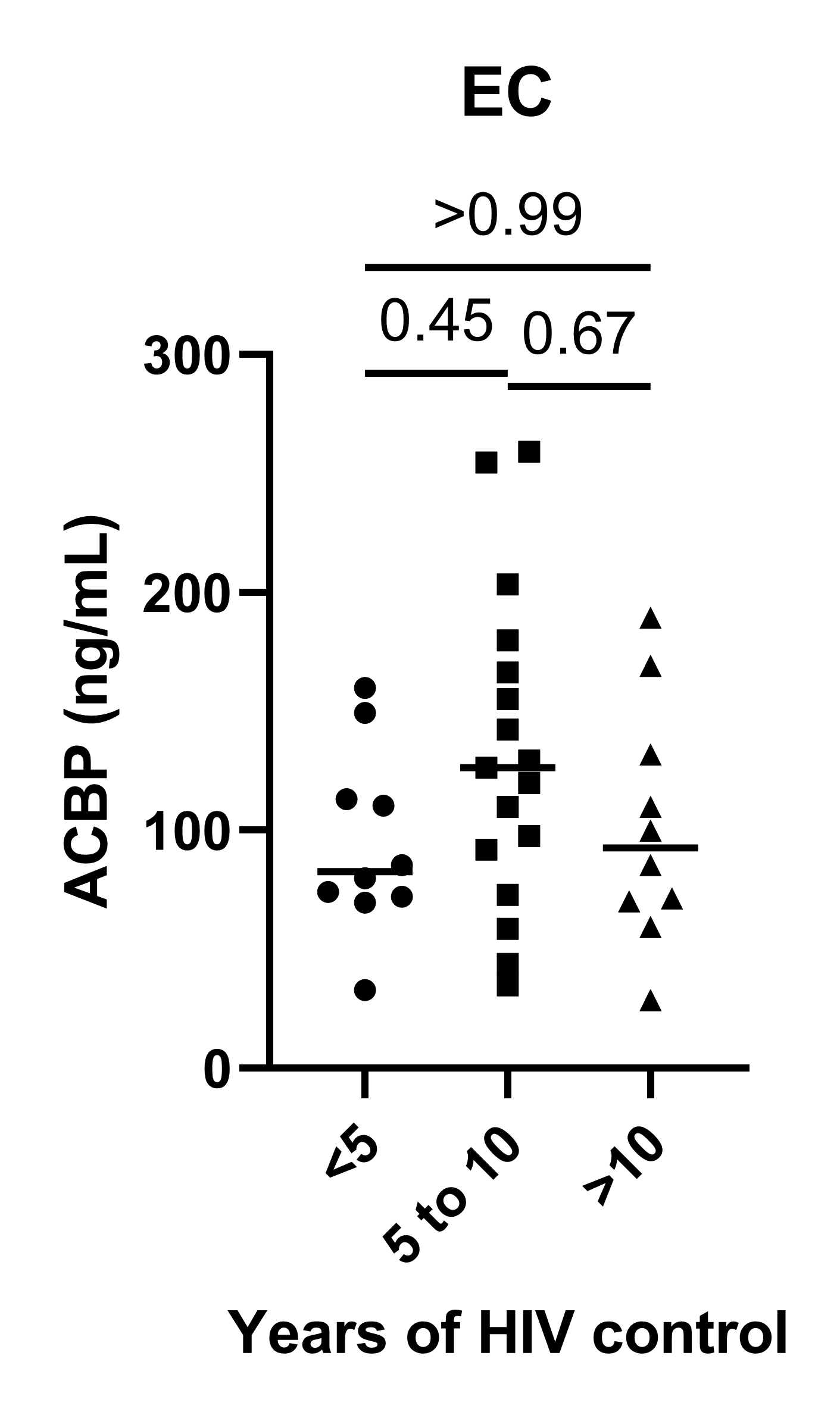

Supplement: Supplementary file 1 [file viruses-14-00453-s001.zip › Fig S1.tif]

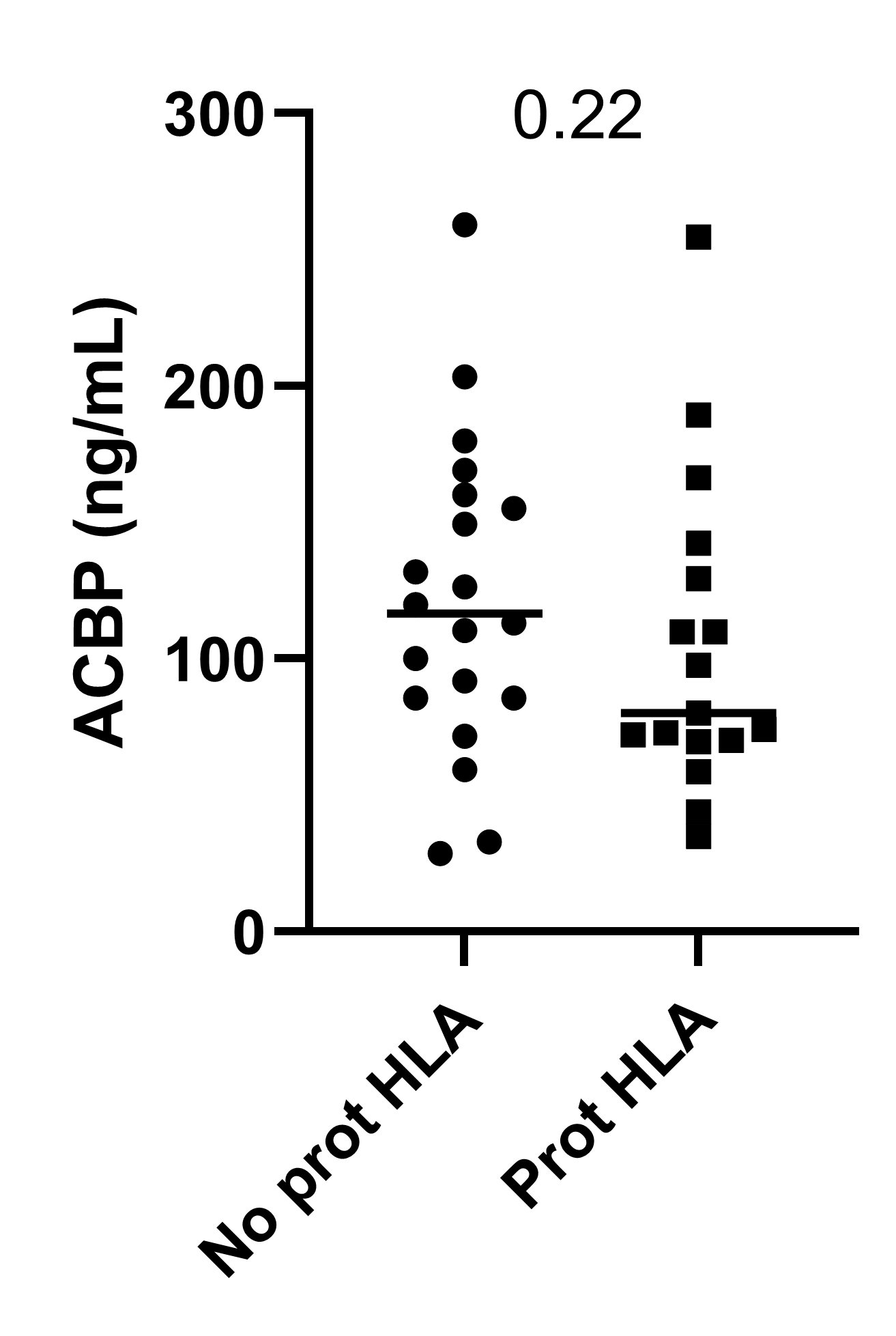

Supplement: Supplementary file 1 [file viruses-14-00453-s001.zip › Fig S2.tif]

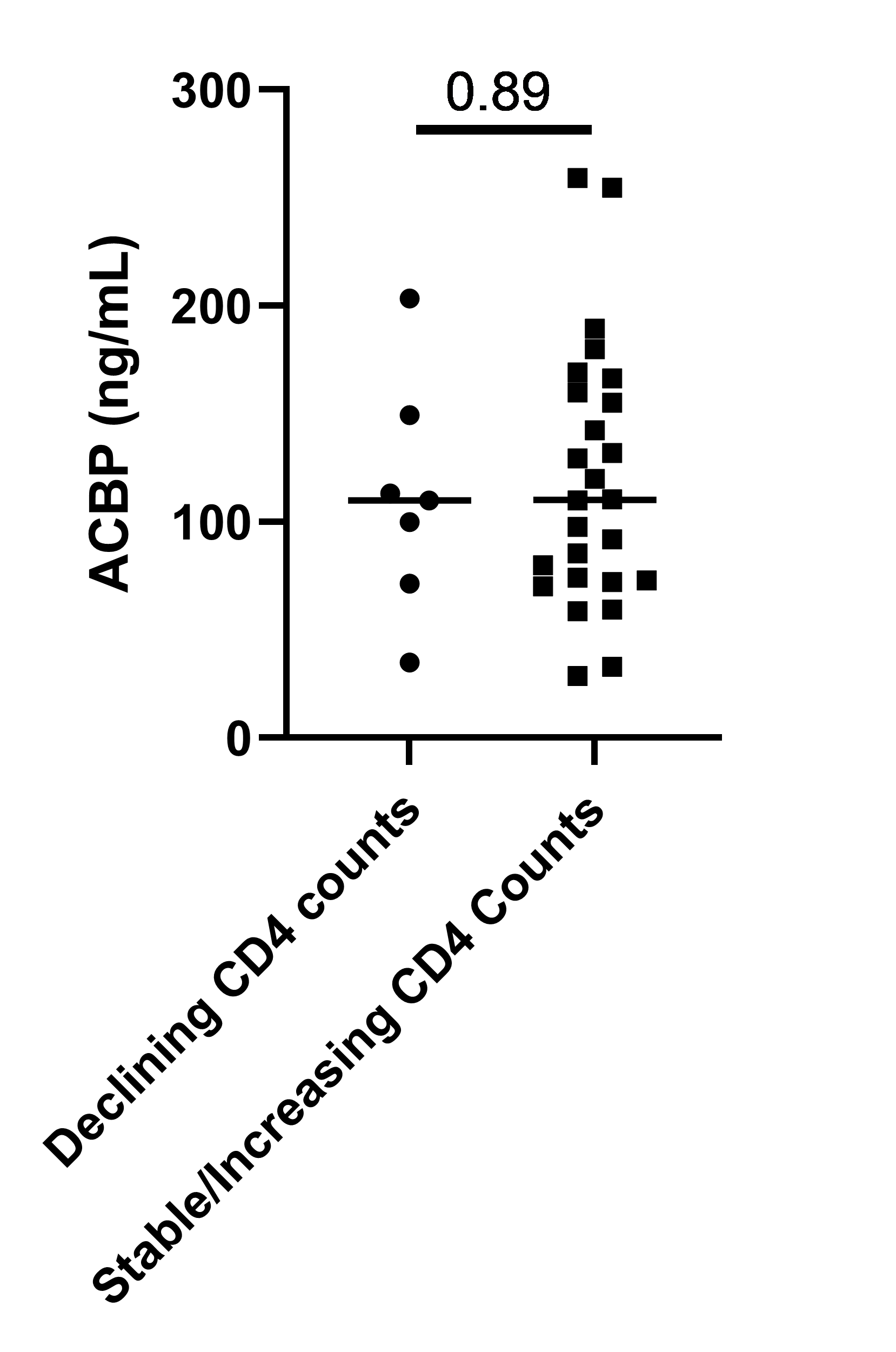

Supplement: Supplementary file 1 [file viruses-14-00453-s001.zip › Fig S3.tif]

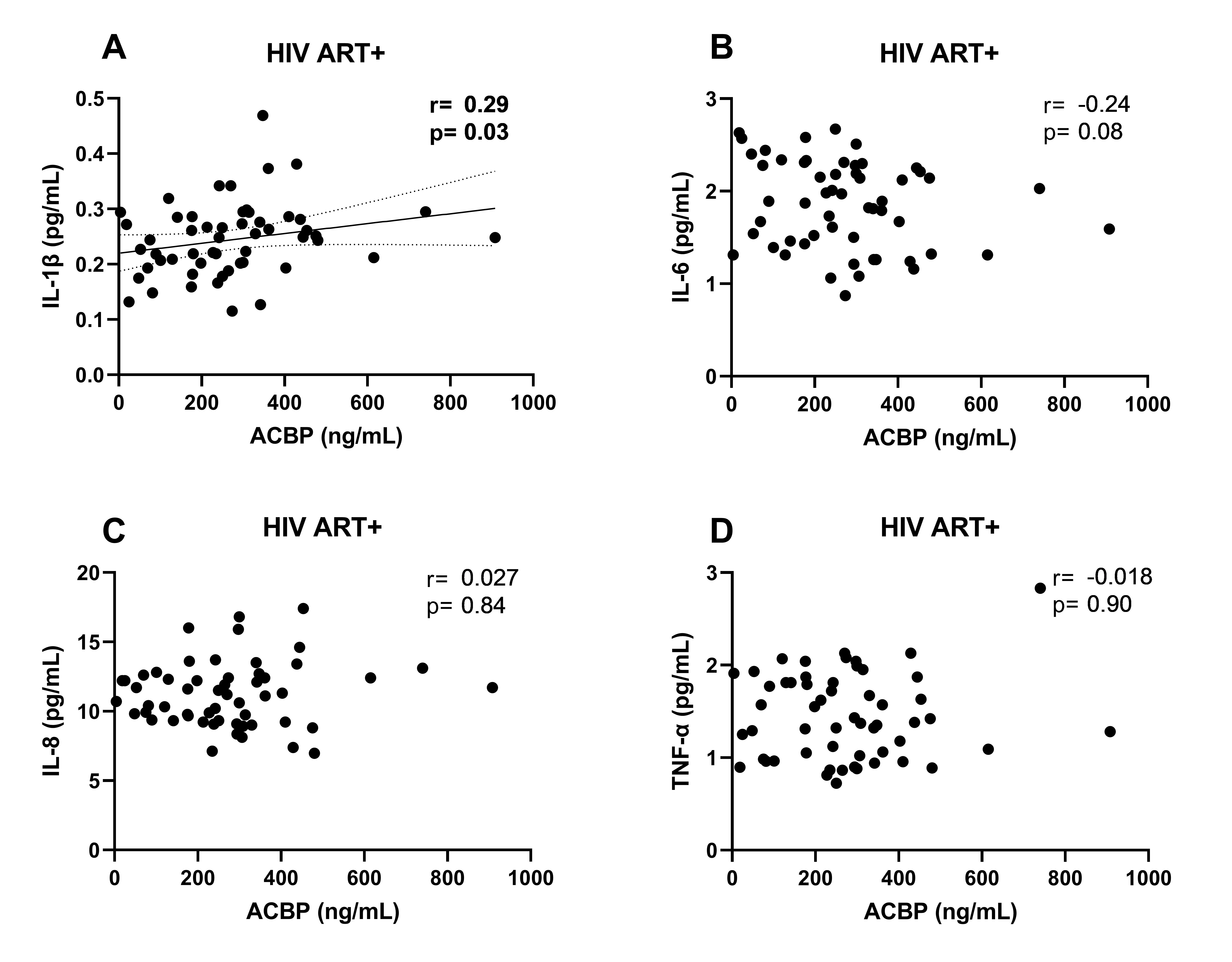

Supplement: Supplementary file 1 [file viruses-14-00453-s001.zip › Fig S4.tif]
